# Natural Rubber Biocomposites Filled with Phyto-Ashes Rich in Biogenic Silica Obtained from Wheat Straw and Field Horsetail

**DOI:** 10.3390/polym13071177

**Published:** 2021-04-06

**Authors:** Marcin Masłowski, Justyna Miedzianowska, Maciej Delekta, Agnieszka Czylkowska, Krzysztof Strzelec

**Affiliations:** 1 Institute of Polymer & Dye Technology, Lodz University of Technology, Stefanowskiego 12/16, 90-924 Lodz, Poland; justyna.miedzianowska@dokt.p.lodz.pl (J.M.); 218652@edu.p.lodz.pl (M.D.); krzysztof.strzelec@p.lodz.pl (K.S.); 2 Institute of General and Ecological Chemistry, Lodz University of Technology, Zeromskiego 116, 90-924 Lodz, Poland; agnieszka.czylkowska@p.lodz.pl

**Keywords:** phyto-ash, biogenic silica, horsetail, straw, natural rubber, biocomposites, functional properties

## Abstract

The rich structural hierarchy of plants permits the obtainment of porous structures which can be expected to show improved performances in fields such as pharmaceuticals and cosmetics, catalysis, drug delivery, adsorption, separation or sensors in various chemical reactions. On the other hand, porous materials can be an active additive to polymer composites. The aim of the study was to obtain natural rubber (NR) biocomposites with the addition of phyto-ashes reach in biogenic silica from plant biomass. For the production of bioadditives, a two-stage method of high-temperature heat treatment was used, preceded by acid hydrolysis of plant tissues in the form of horsetail and wheat straw. Hydrolysis was performed with hydrochloric and citric acid. The efficiency of the processes and their influence on the elemental composition, surface morphology, thermal stability and particle size of the fillers were determined. Modified bioadditives were introduced into the elastomer matrix and their processing properties, as well as the vulcanization characteristics, were examined. Static mechanical properties (tensile strength, elongation at break, stress at 100%, 200% and 300% elongation), dynamic-mechanical analysis and the influence of additives on the cross-link density of the composites were determined. Structural analysis was performed using scanning electron microscopy. It was found that the field horsetail and cereal straw are plants rich in many valuable chemical compounds, especially silica. The specific and appropriate treatment of these plants can lead to bioadditives that significantly affect the properties of rubber materials.

## 1. Introduction

Nanotechnology is a field of study, where the matter is used for industrial purposes on the nanometer scale [1]. Nanotechnology is a branch of science widely developing in the field of materials engineering [2], especially with the use of natural resources [3,4]. Silicon as an element has plenty of interesting properties. It is widespread around the world. Soil consists of 32% of silicon by weight [5]. Silicon dioxide has numerous applications. It is used as a filler for concrete [6], in the pharmaceutical industry, as a catalyst or in medicine as a drug delivery agent [7]. Silicon dioxide can also increase bone density and help with the regeneration of certain tissues [1]. Moreover, silica nanoparticles are biocompatible and were used in cancer treatment [1].

The sol-gel synthesis method is often used for producing silica nanoparticles [6]. However, cleaner solutions can be achieved. Green chemistry is an eco-friendly alternative method that is focused on achieving sustainability [8]. Lately, more and more materials are obtained from natural resources, such as polyethylene [9]. Although silica is present commonly in nature there is no biochemical reaction that would require silicon [10]. However, silica can be used as a strengthening agent in some crops [11], and because of that, some plants might be a great source of silicon dioxide with a nanometer scale and much more ecological than older methods [6]. Such silica has a big surface area, that can reach up to several hundred square meters per gram [7]. However, silica gained in this way has irregular shapes [6].

A great example of such a source is horsetail, which contains up to 25% silica by weight [10]. Most of this silica is in the secondary cell wall [12]. Horsetail is a vascular plant. The content of silica in *Equisteum* may differ by its origin [7]. Other substances appearing in Horsetail are proteins, lipids, cellulose, metal ions like potassium, calcium, sodium and aluminum [12]. Characteristics of biogenic silica strongly depend on processing and pre-treatment of the crop [7]. It can be found in Asia, North America and also in Europe [13]. Other important characteristics of this plant are their rhizomes, which can reach a depth of one meter or more. Apart from that, it has incredible abilities to regenerate. The new plant can grow from just 3 cm cut fragments [14]. Those traits make horsetail hard to remove from fields.

Another crop that contains big amounts of silica in cells is the *Gramineae* family. Most commonly used for deriving silica is rice and especially its husks. Wheat, as a representative of the same family also can be used for extracting this substance [5]. Wheat straw can be purchased cheaply from local farmers. It is commonly used as litter for animals. It is composed of 8–15% lignin, 20–30% hemicellulose and 35–45% cellulose [15]. Apart from biogenic silica, cellulose nanofibers could be also obtained from wheat straw [16]. However, acid treatment can also lead to some by-products such as formic acid, furfural, acetic acid and 5-hydroxymethylfurfural. This is especially unwanted while processing cellulose to biodiesel [17]. As a replacement for acid ionic liquid can be used for dissolving cellulose [18]. Rice straw is another great source of biogenic silica. Scientists could gain pure silica in the form of nano-disk by some exact heating conditions and further treatment [19]. It is also possible to get biodiesel from lignocellulose. It could be obtained from corn, wheat and rice leftovers [17].

Natural rubber is a composition of 94% cis-polyisoprene, and 6% other compounds, such as carbohydrates, lipids and proteins [20]. Some of those naturally occurring proteins cause a type 1 allergy, that can be potentially lethal for humans. However, new techniques of removing the allergenic proteins were developed [21]. Proteins and lipids were found to occur only on the surface of the rubber core. This surface is about 20 nm thick. Presence of lipids and other chemicals in rubber latex improves quality and overall performance of final rubber vulcanizates [20]. It clearly shoves importance of research on naturally obtained materials.

Rubber compounds are being filled with dozens of different fillers to achieve improvement of quality as well as cost reduction per unit. Silica is often used in such compounds. It was proven to increase tear strength, decrease rolling resistance while using it as a material for tires production [22]. Apart from silica, carbon black is the second most used filler in NR composites. It enhances such characteristics as a modulus, abrasion resistance and others related to strength [23]. As for different composites of NR, some are based on fibres, such as banana fibre [24], sisal and oil palm. Wheat straw can also be used as a filler for some polyesters after steam treatment [25]. The natural rubber has usage in such tires. Interestingly bamboo fibre was also used as an ingredient for rubber compound, which improved tensile module and hardness [26]. NR is usually obtained from *Hevea brasilensis* [27]. This plant, apart from producing latex mixture has, other interesting appliances. One of them was to make biodiesel out of its seed oil [28]. The interest in composites based on natural rubber is more and more developed, but still less than in resin or thermoplastic composites. The properties of the reinforced composites are not merely based on the properties of matrix or reinforcing particles, there are other parameters that contribute to the functional properties improvement, which mainly influence the outcome of the composite material. The parameters considered in this research are the volume fraction (amount), size of the particle and the interface particle/matrix, dispersion of additives in the polymer [29]. Due to the important role of additive particles in polymer composites, it is fundamental have full knowledge of the fillers nature and properties.

Some plants, as for example wheat, horsetail or rice, are great source of important materials and compounds. In previous works, the authors investigated the effect of using lignocellulosic materials such as straw (various types) [30,31] and field horsetail [32] as fillers of natural rubber vulcanizates (NR). In addition, many methods of straw modification have been presented to increase the adhesion between the fibre and the polymer matrix [33,34,35,36]. Moreover, natural extracts of horsetail, acting as anti-aging agents, were also used as additives to natural rubber [37]. The novelty of this work is the use of plant tissues (wheat straw and horsetail) for the production of fillers for elastomer composites in the form of silica-rich phyto-ashes.

In this study authors focused on obtaining natural rubber compound filled with biogenic silica, so both materials may be obtained from natural, renewable sources. To the best of our knowledge, it was never obtained in a such way. Apart from that, silica obtained by different methods were characterized by various methods. Materials, such as natural rubber filled with biogenic silica, can be interesting alternative for conventional ones, that require conducting often complicated and wasteful procedures.

## 2. Materials and Methods

### 2.1. Materials

Field horsetail (HT) was delivered by MANU JTC, s.r.o. (Opawa, Poland). Common wheat (WS) was harvested from local farms. Hydrochloric acid (Sigma-Aldrich, Schnelldorf, Germany) and citric acid (Sigma-Aldrich, Schnelldorf, Germany) were used in the hydrolysis process. The polymer matrix was natural rubber (NR) supplied by Torimex-Chemicals Ltd. Sp. z.o.o (Konstatntynów Łódzki, Poland). The cross-linking system consisted of sulfur (Siarkopol, Tarnobrzeg, Poland), 2-mercaptobenzothiazole (MBT), purchased from Sigma-Aldrich (Schnelldorf, Germany), micrometric zinc oxide (ZnO, Huta Będzin SA, Będzin, Poland) and stearin (SA, Avantor Performance Materials, Gliwice, Poland).

The composition of the rubber mixture included (Table 1): natural rubber NR (100 phr-parts per hundred rubber), zinc oxide (5 phr), sulfur (2 phr), 2-mercaptobenzothiazole (2 phr), stearin (1 phr) and a filler (0 phr in in the case of the reference sample and 10 phr in the case of samples filled with the tested bioadditive).

### 2.2. Methods

The dried plant material was ground in a planetary ball mill and then subjected to additional drying at 70 °C for 24 h. Then, for 50 g of plants, acid hydrolysis was performed in 1 M hydrochloric acid and 10% citric acid for 2 h at the boiling point of the solution. After cooling, the samples were drained and washed several times with water. The next stage was burning the sludge at 700 °C with air access. High-temperature treatment was carried out for 5 h.

The thermal decomposition of additives was investigated by applying TGA thermogravimetric analysis. The sample was heated at 10 °C/min in the range from 25 °C to 800 °C in an air atmosphere. A curve of the percentage weight loss versus temperature and its derivative were determined. Test was carried out using a TGA analyzer (Mettler Toledo, Greifensee, Switzerland).

DLS light scattering analysis was used to determine the size of the obtained filler particles. 0.02 g of the tested material was dispersed in 1 dm^3^ of distilled water by the action of ultrasound for 0.5 h. The colloid was placed in a polypropylene cuvette and analyzed by DLS on a Zetasizer (Malvern Panalytical Ltd., Malvern, UK).

The composition and purity of the fillers were determined by means of FTIR infrared spectroscopy with the use of light in the range 400–4000 cm^−1^.

The content of Mg, Ca, Al, Fe and Si in solid compounds were determined by the ICP-OES: PlasmaQuant PQ 9000 Elite, Analytik Jena (Jena, Germany). Limit of quantification were Mg: 2 ug/L, Ca: 2 ug/L, Fe: 7 ug/L, Al: 20 ug/L, Si: 50 ug/L. Solid samples were digested in the mixture of concentrated acids (1 mL of 36% HCl and 6 mL of 65% HNO_3_) decomposed using the Anton Paar Multiwave 3000 (Graz, Austria) closed system instrument. Mineralization was carried out for 45 min at 240 °C under pressure 60 bar.

The composition was additionally examined using atomic absorption spectroscopy and scanning electron microscopy with an EDS attachment (Hitachi, TM-1000, Tokyo Japan). Additionally, SEM images were used to study the morphology of the obtained fillers and vulcanizates. SEM images of elastomeric materials were taken from fractures of the cryogenically processed samples.

The components of the rubber compounds were weighed on an analytical balance in accordance with the amounts given in Section 2.1. The mixes were made on a Bridge, Rochdale, UK laboratory rolling mill. Process parameters: roll dimensions L = 450 mm, D = 200 mm; rotational speed of the front roller Vp = 16 rpm; width of the gap between the rolls 1.5–3 mm; friction 1–1.2; average temperature of the rolls 30 °C; mixing time 10 min.

The measurement of rheometric properties was performed at 160 °C based on the PN-ISO 6502:2007 standard, on an MDR rheometer (Alpha Technologies, New York City, NY, USA). The minimum torsional moment (M_min_), maximum torque (M_max_), torque increase (ΔM), scorch time (ts_2_), and vulcanization time corresponding to 90% of the maximum torque (t_90_) were read from the vulcametric curve.

In order to check the functional properties of the obtained fillers, elastomer composites were made using a hydraulic press. The process was carried out at a temperature of 160 °C with a pressure of 15 MPa. The time was selected based on the previously obtained parameters.

The cross-linking density (γ_e_) was determined by the equilibrium swelling method at room temperature. Toluene was used as a solvent. The study was conducted on the basis of the PN-74/C-04236 standard. The density value was calculated from the Flory Rehner equation [38].

(1) $$\gamma_{e}=\frac{\ln\left(1-V_{r}\right)+V_{r}+\mu V_{r}^{2}}{V_{0}\left(V_{r}^{\frac{1}{3}}-\frac{V_{r}}{2}\right)}\;$$

where: 𝛾_𝑒_—concentration of effective chains, *V_r_*—volumetric fraction of rubber in the swollen gel, *V*_0_ molar volume of the solvent [mol/cm^3^], *μ*—Huggins parameter (elastomer-solvent interactions), determined at 25 °C:

(2) $$\mu_{NR+t}=0.478+0.228\cdot Vr\;$$

*V_r_* is the volume of the elastomer fraction in the swollen sample:

(3) $$V_{r}=\frac{1}{1+Q_{w}\frac{\rho_{k}}{\rho_{r}}}\;$$

where: *Q_w_*—swelling reduced by the filler content (x [phr])—*Q_w_* = (100 + x/100), *ρ_k_*—rubber density [g/cm^3^], *ρ_r_*—solvent density [g/cm^3^].

The study of mechanical properties in static conditions was carried out based on the ISO-37 standard on a testing machine with an extensiometer (Zwick, Ulm, Germany). The paddle-shaped samples were stretched at a speed of 500 mm/min. The relative elongation at break, tensile strength, and stresses at 100%, 200% and 300% relative elongation were determined for 5 specimens, and then the average value was determined.

The dynamic mechanical properties test was carried out on an Ares G2 Rhometer (TA Instruments, New Castle, UK). Parameters: tension 0.1–150%; defomation rate 10 rad/s; pressing force 5 N; temperature 25 °C. The Payne effect was determined on the basis of registered changes of the storage modulus as a function of dynamic deformation.

A DMA/SDTA861e (Mettler Toledo, Greifensee, Switzerland) analyzer was used to test the viscoelastic behavior of the NR vulcanizates filled with phyto-ashes as a function of temperature. The measurements were performed in the temperature (T) range of −120 to 60 °C with a heating rate of 3 °C/min. Shape of the test specimens was: length—10.5 mm, width—4 mm and thickness—1 mm. Samples were stretched via oscillation with a frequency of 1 Hz and a strain amplitude of 4 µm. The glass transition temperature (Tg) of natural rubber vulcanizates was determined on the basis of the maximum of tanδ = f(T) plot, where tanδ is the loss factor.

## 3. Results and Discussion

### 3.1. Efficiency of the Hydrolysis Process and High-Temperature Treatment

On the basis of mass measurement before and after the acid hydrolysis and high-temperature treatment, the yields of individual stages were calculated. The results are summarized in Table 2. Acid hydrolysis, depending on the type of acid and material, removed from 22.5% to 44.1% of dry solids. Hydrolysis is a process that has been used in these studies for several purposes. The first was the reduction of lignocellulosic material. Acid hydrolysis is mainly responsible for the removal of hemicelluloses [39]. To produce high-quality biogenic silica from plant parts, it is necessary to remove the remaining inorganic matter before heat treatment. Otherwise, crystalline silica will form. This is because alkaline ions such as sodium, potassium and calcium catalyze the phase transition from amorphous to crystalline silica, lowering the temperature of this process [40]. In addition, this process may be accompanied by carbonization, that is, the entrapment of carbon, which cannot be completely removed later [41,42]. Acid hydrolysis with hydrochloric acid was more effective (E1) than that carried out with citric acid. The difference was especially noticeable in the case of straw hydrolysis. The efficiency of the process for the treatment of horsetail was at a similar level, regardless of the acid used. The amount of matter removed from straw ranged from 43% to 20% with hydrochloric acid and citric acid, respectively.

On the other hand, high-temperature treatment resulted in a significant reduction of the material, along with the thermal degradation of organic compounds. It was mainly cellulosic and lignocellulosic material. Plant samples that had not previously been hydrolyzed left much larger amounts of material after burning. This is evidenced by the reduced yield of the second stage (E2) obtained for non-hydrolyzed samples compared to those treated with acid treatment. It was the effect of removing basic compounds of elements such as calcium or potassium, which could not be removed from plants only by burning them [12,15].

### 3.2. Thermogravimetric Analysis

**Figure 1:**
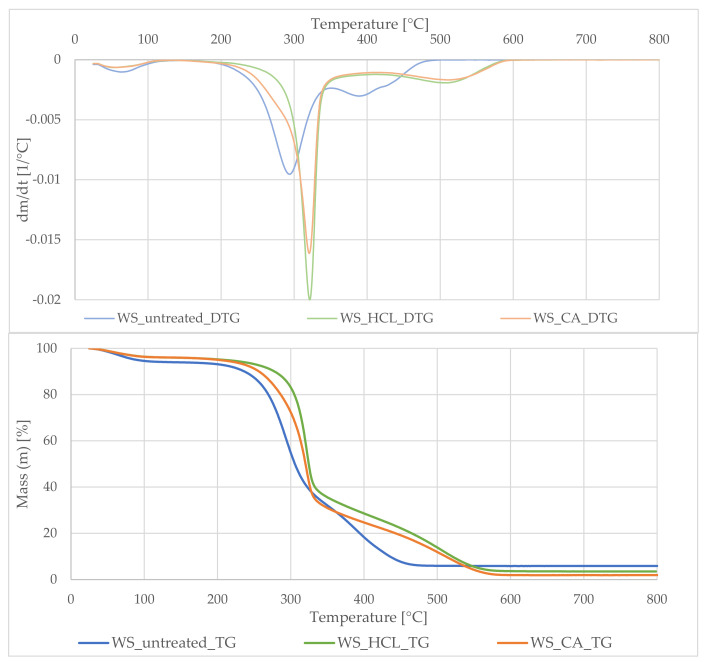
The thermogravimetric (TG) curves (**down**) and the derivative of the TG versus time (DTG) curve (**up**) of wheat straw untreated and treated with acids.

**Figure 2:**
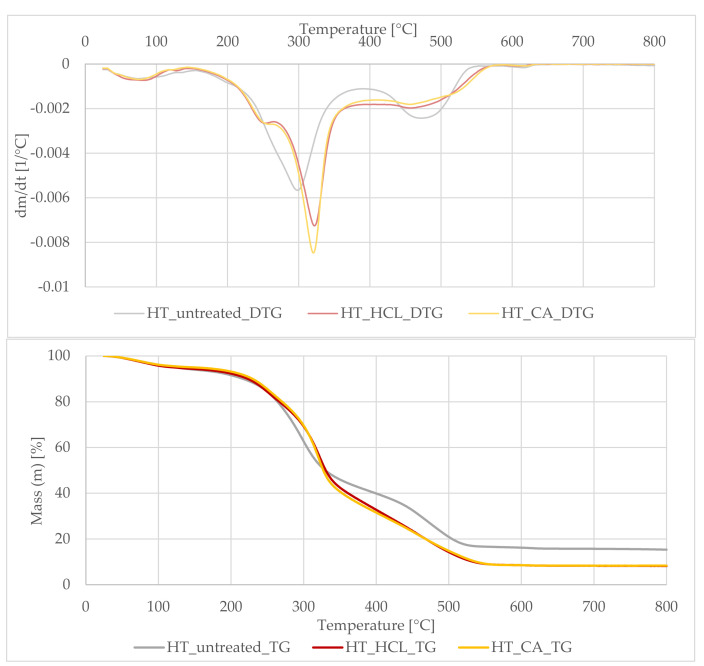
The thermogravimetric (TG) curves (**down**) and the derivative of the TG versus time (DTG) curve (**up**) of horsetail untreated and treated with acids.

Based on the thermogravimetric analysis, it was determined what processes take place during high-temperature processing of plant material. The analysis of the graphs in Figure 1 and Figure 2 indicated that the processes taking place in both materials took place in 3 stages. Changes between samples in weight loss and process intensities in given temperature ranges resulted from different content of the main plant components and acid treatment, influencing the quantitative and qualitative composition of materials.

The first stage, marked on the DTG curve with a peak ranging from 50–100 °C, was related to the evaporation of free water absorbed by plant samples. In the case of the HT_untreated sample analysis, the decomposition took place in a slightly wider temperature range and was extended to 160 °C, it was caused by a high content of volatile compounds such as flavonoids, phenolic acids, and dyes contained in horsetail. The subsequent decomposition steps in the temperature range 200–350 °C and 350–550 °C were closely related to the decomposition of individual building blocks of plants: lignin, hemicellulose and cellulose. Lignin decomposes over a very wide temperature range, from 150 °C to 650 °C [43]. On the other hand, for the hemicellulose material, Shen observed two significant weight losses, the first from the temperature of 198 °C with the highest intensity at 263 °C, while the second, related to the decomposition of carbon residues at 440 °C [44]. According to Yang [45], the degradation of cellulose is concentrated in the narrower temperature range of 315–400 °C. In the third stage (400–550 °C), apart from further decomposition of the lignocellulosic material, decomposition of its charred residues took place. Comparing the data obtained for the hydrolysed samples, a smaller change in mass was clearly observed during the decomposition of carbonaceous residues. The acid hydrolysis of hemicellulose comprised two stages, solubilization and partial destruction of the reducing sugar formed [46]. Therefore, during firing, there was less weight loss of the sample. Moreover, it was noted that the last step for the hydrolyzed materials was shifted towards higher temperatures. This was due to the fact that the acid hydrolysis process significantly reduced the content of alkaline ions in plant samples. According to Adach et al. [7], these accompanying ions can reduce the calcination temperature of samples by 100 °C. The same effect was observed in the presented studies. Moreover, these ions have a catalyzing effect on the carbonization reaction [41]. Consequently, the high content of alkali ions of calcium, sodium, potassium, etc. during calcination contributed to the degradation of the SiO_2_ spatial structure, resulting in sintering of silica particles with others.

In conclusion acid hydrolysis removed the portion of the organic material of plant tissues. Treating the fillers beforehand resulted in a cleaner end material. The above analysis confirmed the validity of the hydrolysis step.

### 3.3. Analysis of Filler Composition Using ICP-OES Technique

Plant macronutrients such as calcium and magnesium, as well as trace elements such as aluminum and iron, were studied in all samples (Table 3). Additionally, the content of silicon and solid residues after mineralization were determined. Calcium and magnesium are important ingredients in plant nutrition. There is selective bioaccumulation of these elements in plants. As macronutrients, they occur in plants in much greater amounts than other metals. Calcium is part of the building material of plants and is responsible for the structural stability of tissues, while magnesium stimulates the development of the root system and activates the processes responsible for the uptake of minerals from the soil. It should be noted that WS is definitely poorer in the determining elements. After treating WS and HT with citric and acetic acids, respectively, the contents of these elements decrease drastically. The reduction in the content of these metals is due to the formation of soluble in water metal citrates and metal chlorides, respectively. These compounds are present in the filtrates after washing the samples. The situation is different in the case of the deter-mined micronutrients. The contents of iron and aluminum for WS and HT are in the range of 0.03–0.6 mg/L. After hydrolysis in acids, their concentrations do not change much. This is due to the very low contents of these elements in plants. Much higher than wheat straw silicon content in horsetail is due to the characteristics of this plant. This content (1.78 mg/L) decreases for HT_CA and HT_HCl, which is related to the transfer of some of the silicon compounds into the acids solutions. In the case of WS, this trend is increasing. This is probably due to the presence of other forms of silicon compounds characteristic of WS. Analyzing the solid residues remaining after mineralization, in both cases, we can see increasing values of the probably formed silica.

### 3.4. Determination of the Particle Size of Fillers Using the DLS Method

The size and development of the surface are very important parameters in the case of fillers for elastomer composites. They play an important role in strengthening the elastomeric material. The size of the obtained phyto-ash particles was determined to assess whether they could be classified as nanomaterials. Moreover, the size of the filler particles has a significant influence on the final properties of elastomer composites. Filler particles with the size of nanomaterials are usually characterized by a developed specific surface, which means that the surface of polymer-filler interfaces is also large, which has a positive effect on the strengthening effect. Primary particles of the filler in a non-agglomerated form are extremely rare under real conditions. As a result of the physicochemical interactions of a few to a dozen primary particles of the filler, aggregates are formed. Excessive tendency of the filler to form aggregates and then agglomerates in the polymer matrix results in deterioration of the multifunctional properties of composites.

**Figure 3:**
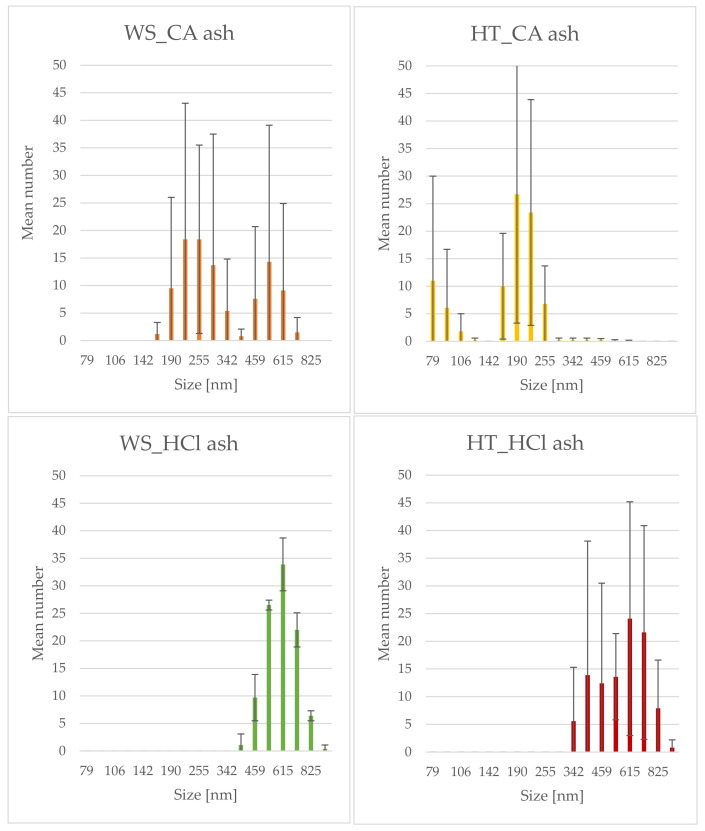
Size of the fillers obtained by acid hydrolysis and high-temperature treatment of plants.

The dynamic light scattering method was used to determine the particle size of the obtained phyto-ashes. The results of the analysis are presented in the form of histograms illustrated in Figure 3.

By analyzing the above histograms, it was found that phyto-ash particles obtained from plants hydrolyzed in hydrochloric acid had a larger size than those hydrolyzed in citric acid. However, the particles previously treated with CA acid had a larger size distribution. This could have been influenced by the undesirable reactions taking place during their preparation. In the case of hydrolysis with citric acid, more basic ions remained in the samples, e.g., calcium, as confirmed by the ICP method. During calcination, the presence of these elements may have contributed to the formation of sintered silica structures with other materials. As a result, the resulting structures were less homogeneous, both in terms of dimensions and shape. The sizes of the particles obtained ranged from about 230 nm to 650 nm. One peak was observed at about 70 nm. According to the definition, the dimensions of nanomaterials should not exceed 100 nm [1]. This means that only some of the agglomerates belonged to these materials.

### 3.5. Analysis of FTIR Spectra

**Figure 4:**
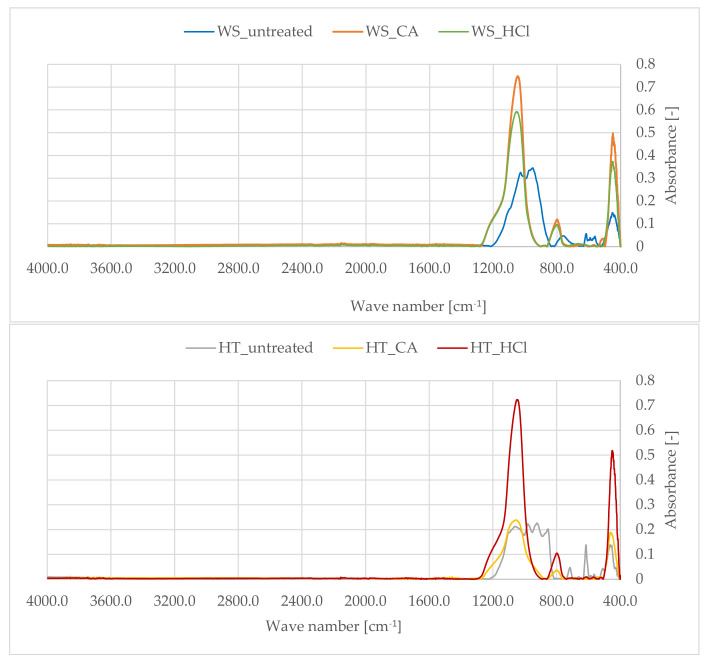
FTIR spectra of plants ashes from wheat straw (**up**) and horsetail (**down**).

By analyzing the spectra of infrared spectroscopy with the Fourier transformation, it was possible to obtain information on the structure of the tested samples in terms of the qualitative composition of the materials. In the case of silicon dioxide, the typical spectrum is characterized by the presence of three bands with maximum peaks at 1100, 800 and 450 cm^−1^ [47,48]. For all samples of the tested phyto-ashes, analogous maxima were obtained, differing in their surface areas (Figure 4). The maximum at the wavenumber of 450 cm^−1^ corresponds to the asymmetric vibrations bending silicon-oxygen bonds. The peak for the wavenumber 800 cm^−1^ corresponds to symmetrical vibrations stretching the Si-O bond. The highest peak in the spectra, in the wavenumber range 900–1300 cm^−1^, corresponds to asymmetric vibrations stretching silicon-oxygen bonds. The conducted FTIR measurements confirmed the high silica content in the obtained phyto-ashes from wheat straw and field horsetail.

Both the spectra obtained for the phyto-ashes of wheat straw and horsetail had analogous maxima, however, the signals obtained for the non-hydrolyzed samples clearly differed from the others. For these phyto-ashes (WS and HT), the maximum values of Si-O bonds corresponding to vibrations were smaller and slightly shifted in relation to their counterparts in the other spectra. Signals from these vibrations were jammed by others from pollution. Nevertheless, they were still perfectly visible. Comparing the spectra of non-hydrolyzed ashes, many smaller peaks from pollutants were also observed. Contamination could come from compounds not leached out by hydrolysis and baked with silica in the material. The FTIR analysis allowed to confirm the validity of the hydrolysis stage of plant tissues in order to obtain the purest biogenic silica.

### 3.6. SEM-EDS Analysis

**Figure 5:**
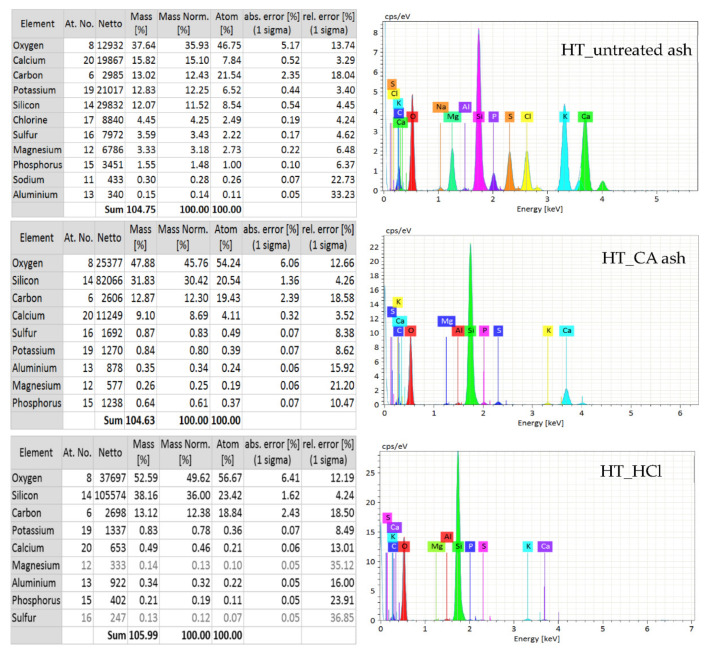
Elemental composition of horsetail ashes.

**Figure 6:**
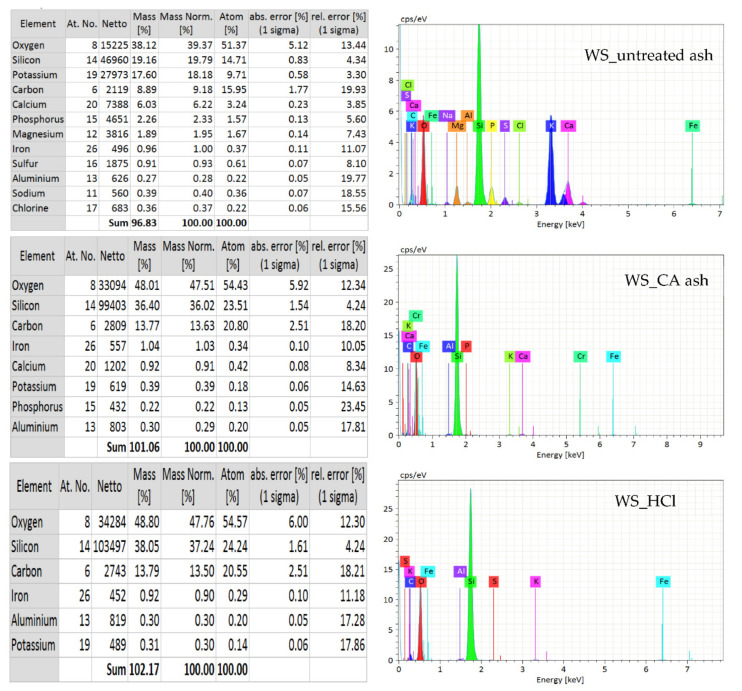
Elemental composition of wheat straw ashes.

Scanning electron microscopy allows to determine the morphology of the examined surfaces. Additionally, the EDS (Energy Dispersive Spectroscopy) attachment is used to analyze the elemental composition. Therefore, it was possible to confirm large amounts of silica. The results of the SEM-EDS analysis are shown in Figure 5 and Figure 6. Hydrochloric acid was more effective at removing calcium ions during hydrolysis, but the action of both acids was very similar. The silicon-derived signal was significantly greater than that of the rest of the elements, both in the hydrolyzed and the non-hydrolyzed sample. A large signal was also observed for oxygen, indicating that most of the observed elements were in the form of oxides.

Most of the basic substances were removed by acid hydrolysis. The SEM-EDS analysis additionally indicated that the non-hydrolyzed ash contains elements such as Ca, K, Cl, S, P and Mg. Wheat straw ash contains less magnesium, while the iron signal is additionally marked.

**Figure 7:**
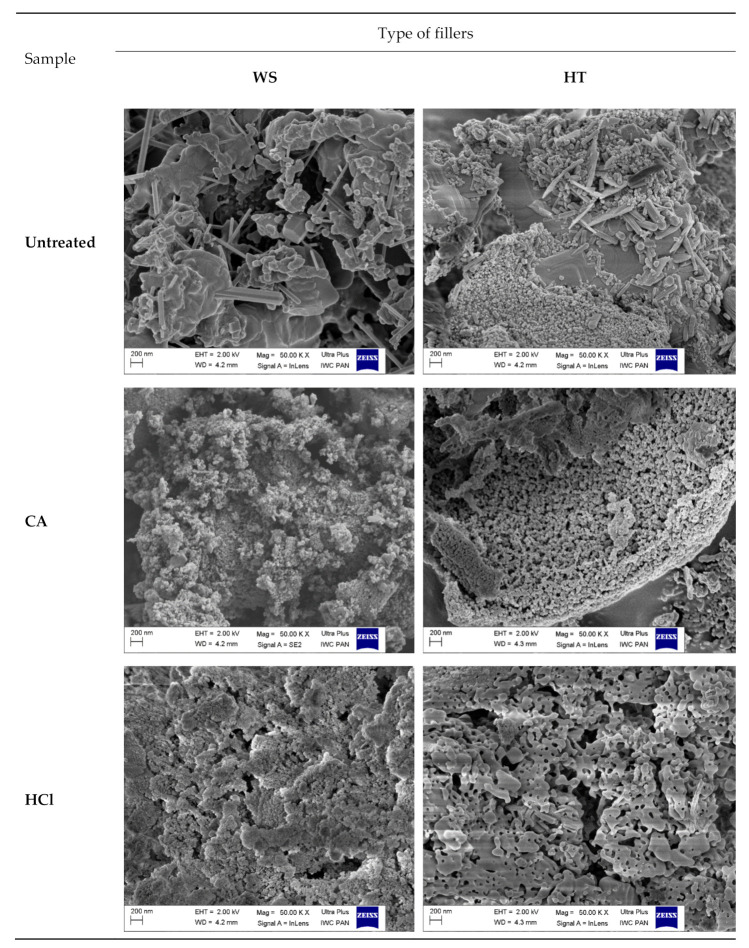
SEM images of phyto-ashes.

The analysis of SEM images of the ashes is presented in Figure 7. The surfaces of the fillers subjected to acid hydrolysis were much more uniform. Moreover, the previously hydrolyzed samples showed a porous morphology. In the case of phyto-ash from untreated plants, structures of various shapes and dimensions were observed in the images. Both spherical particles (especially HT_untreated samples) were present, but also hexagonal wire particles were abundant. They can have an additional strengthening effect on the composites. These differences also showed that the hydrolysis step made it possible to obtain much more homogeneous samples. Nevertheless, the nanoparticles accumulated into aggregates and agglomerates. The formation of mesopores that result from the non-condensed agglomeration of SiO_2_ particles depends on the calcination procedure [49]. The obtained structure may be influenced by temperature and the presence of certain ions. The increase in temperature is favored by the increase in particle agglomeration. The specific surface area has been shown to decrease rapidly for samples calcined at 700 °C or higher [50]. On the other hand, impurities can affect the sintering and bonding of particles of various compounds, consequently creating different structures. High content of basic compounds (e.g., K_2_O) can lead to the formation of cristobalite, quartz and tridymite [51]. These phenomena may explain the presence of various structures in non-hydrolyzed samples, which were more diverse in terms of their qualitative composition (Figure 7).

### 3.7. Analysis of Rheometric Properties of Rubber Mixtures Containing Phyto-Ashes

**Figure 8:**
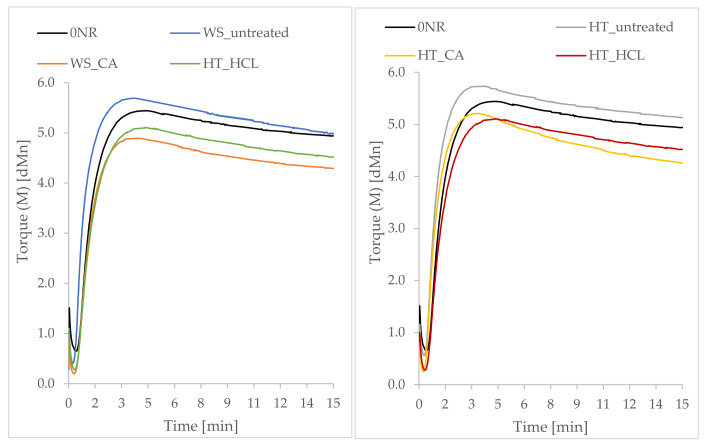
Rheometric curves of rubber mixtures.

The test results of the vulcanization process and the course of the vulcametric curves are presented in Table 4 and Figure 8 respectively. The measure of the mixture viscosity is the value of the minimum rheometric moment. Comparing the obtained values with the reference sample (unfilled natural rubber), it can be concluded that the mixtures containing plant ash had a lower viscosity. Moreover, the viscosity of the mixtures with non-hydrolyzed samples was significantly higher than that of the hydrolyzed samples for both wheat straw and horsetail. This was probably due to the morphology that could be observed in the SEM images.

As shown by the rheometric curves (Figure 8), as a result of the cross-linking reaction, the torque value increased until the maximum torque (M_max_) was obtained. Vulcanization reactions lead to the formation of several different cross-linking structures, including mono-, dis- and polysulfide bonds [52]. In this vulcanization process, after reaching the maximum torque, there was a slight decrease in the M value. This phenomenon is called reversion, which is very common in natural rubber systems [53]. Generally, during reversion of a vulcanizate, the breakdown of cross-linkages, including those of polysulfides [54]. It is assumed that reversion takes place when the desulfurization reaction is faster than the cross-linking reaction. Moreover, the thermal decomposition of polysulfidic crosslinks also results in the formation of cyclic mono- and disulfides and conjugated dienes/trienes [55]. The increase in rheometric torque during the vulcanization of the mixture is an indirect measure of the degree of cross-linking, since its increase is caused by the appearance of cross-links between the rubber macromolecules.

The highest value of the torque increase (ΔM) was observed for mixtures with ashes which were not hydrolyzed in the first stage of preparation. It was also larger than for the reference sample. In the composition of phyto-ashes, apart from silica, there were also many other substances, some of which probably had properties favoring the creation of cross-links. Acid hydrolysis led to the removal of some basic compounds. These, on the other hand, had a positive effect on the efficiency of vulcanization [56]. The higher content of calcium, potassium and magnesium ions in the untreated ash sample could have resulted in greater reactivity of the cross-linker. As a consequence, it led to an increase in the number of cross-links between the rubber chains contributing also to an increase in the value of ΔM for samples filled with untreated ashes. For mixtures containing a filler made from hydrolyzed samples, these values were lower. The small amount of acid that was not washed away had a negative effect on the networking process. In addition, silica exhibits sorption properties and could accumulate components of the cross-linking unit (in particular the accelerator) on its surface, thus reducing the efficiency of the vulcanization process [57].

Scorch time is another important parameter in the processing of unvulcanized elastomer blends. Longer time (t_05_) means more time for carrying out technological processes, e.g., forming. The use of phyto-ashes as fillers did not significantly affect this parameter. The subculture time for the filled samples ranged from 0.38 to 0.65 min, and for the reference sample 0.60 min.

Based on the results obtained, optimum cure time (t_90_) is calculated as the time required for the torque to reach 90% of the maximum achievable torque. Comparing the determined vulcanization time of mixtures made with the use of phyto-ashes to the reference sample, it was shortened (except for the samples of ashes prepared using acid hydrolysis with HCl). A clear reduction of the t_90_ time was observed for the samples filled with WS_untreated and HT_untreated ashes. This effect was probably the result of a more effective vulcanization process for mixtures containing a filler containing alkaline compounds (prepared without the hydrolysis step).

Silica-rich fillers in rubber mixtures delayed the cross-linking processes of composites. The silanol group on the silica surface could interact with the accelerator and activator [58], leading to a delay in the vulcanization processes and, consequently, extending the optimal vulcanization time (t_90_) [59].

### 3.8. Cross-Linking Density of Obtained Vulcanizates

An important parameter determining the properties of rubber vulcanizates is the cross-linking density. It affects a number of operational parameters of elastomeric materials, such as: mechanical properties, hardness, tear resistance, etc. Its value is determined by the method of equilibrium swelling in a selected solvent. Higher cross-linking density strengthens the vulcanizate, but also reduces its elasticity. Covalent network nodes are formed as a result of the vulcanization reaction. Moreover, as additional physical nodes of the network, interactions of the polymer-filler type can arise and thus affect the development of the spatial structure. The results of cross-linking density of natural rubber vulcanizates filled with phyto-ashes are presented in Table 5.

The use of phyto-ashes obtained from wheat straw and field horsetail as natural rubber nanofillers influenced the determined values of cross-linking density in a different way. In the case of composites containing fillers obtained in a two-stage chemical-thermal treatment of plants, the determined νe values were lower (in the case of wheat straw phyto-ashes) or at the level of the value determined for the unfilled sample (in the case of field horsetail phyto-ashes). Comparing the effect of the type of acid used for the treatment of fillers at the stage of their preparation, a higher cross-linking density was obtained for composites containing: in the case of straw phyto-ash—modified with citric acid, in the case of horsetail phyto-ash—for hydrochloric acid. On the other hand, the application of phyto-ashes from non-hydrolyzed plants resulted in an increase in the concentration of rubber nodes. It was a consequence of the increased activity of the WS_untreated and HT_untreated additives used in the cross-linking process. The obtained results corresponded with the increases in rheometric torque determined during the examination of the vulcanization progress. Metal compounds, ie calcium, magnesium, potassium, which were not washed out during the acid hydrolysis of plants, found in NR_WS_untreated and NR_HT_untreated samples increased the efficiency of the cross-linking reaction, which resulted in increasing the cross-linking density of these vulcanizates. On the other hand, the high content of nanosilica in chemically modified fillers lowered the activity of the cross-linking unit, thus affecting their efficiency.

### 3.9. Payne Effect Analysis

**Figure 9:**
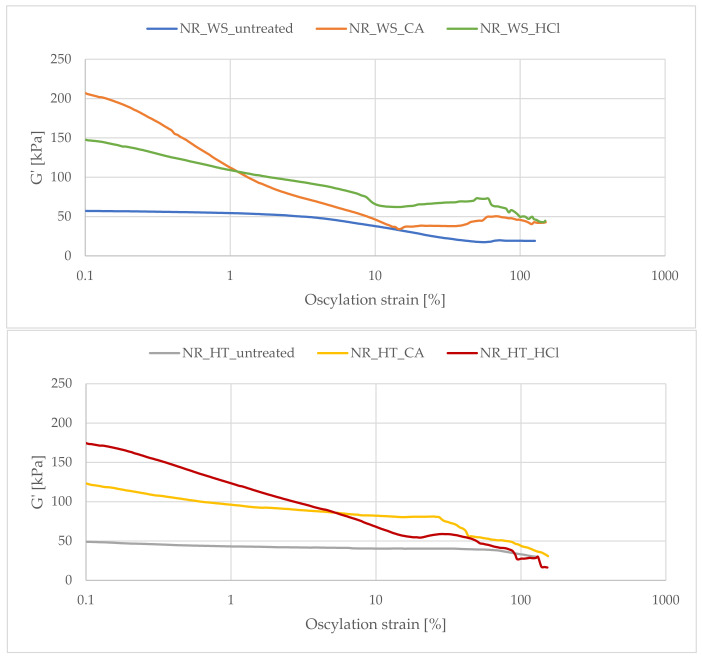
Payne effect (decrease of the storage modulus (G’) as a function of deformation) observed for filled vulcanizates.

In order to determine the Payne effect characteristic of filled elastomeric materials, a study of dynamic mechanical properties was carried out. Figure 9 shows the registered curves of the loss modulus as a function of dynamic strains.

The application to the elastomer matrix of active fillers capable of creating filler-filler and filler-polymer interactions increases the loss modulus G’ and the modulus of elasticity. When deforming such composites, the loss modulus decreases with increasing strain amplitude (Payne effect). This effect was also observed for the tested vulcanizates containing phyto-ashes. The decrease in the G’ module during this test is related to breaking the interactions between the filler particles, i.e., destroying the so-called the secondary structure formed by the filler in the polymer matrix.

Two slope of storage modulus at 10% and 80% were observed on the curves during dynamic mechanical tests. The appearance of these two stages may result from the different nature of bonds that are destroyed during dynamic deformation. It is assumed that the Payne effect is mainly influenced by the destruction of the secondary structure of the filler (filler-filler interactions), and may be affected by other phenomena. These include filler deagglomeration in the framework of selfsimilarity, polymer debonding from filler surface, strain softening of the polymer shell surrounding fillers [60].

Considering the vulcanizates filled with phyto-ashes obtained from wheat straw (Figure 9), the highest value of the storage modulus and its decrease in the deformation function (Payne effect) were observed for the sample containing the filler previously hydrolyzed in citric acid. According to the data on the composition analysis, the filler present in this composite was characterized by the highest amount of silicon compounds. The nanometric silica particles contained in this additive are capable of forming a highly developed filler network which was destroyed during the analysis, resulting in a clear appearance of the Payne effect. In the case of phyto-ashes produced from horsetail, the most developed structure of the filler in natural rubber was observed for the NR_HT_HCl sample. An extensive network of the filler may have a positive effect on the functional properties of composites. However, it should be remembered that too strong filler-filler interactions may lead to an increased tendency to aggregation and agglomeration of the filler, causing undesirable effects.

### 3.10. SEM Analysis of Biocomposites

**Figure 10:**
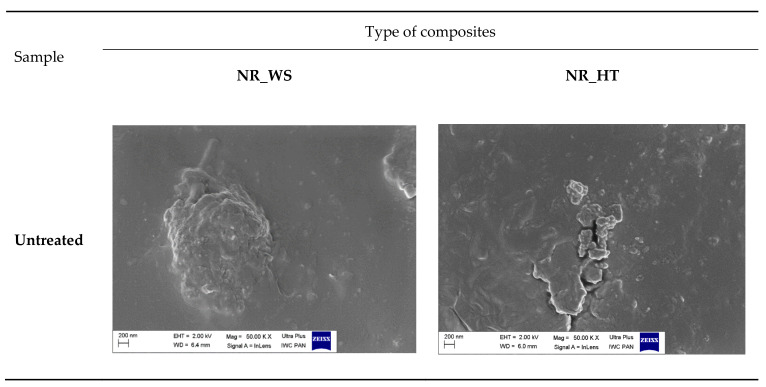

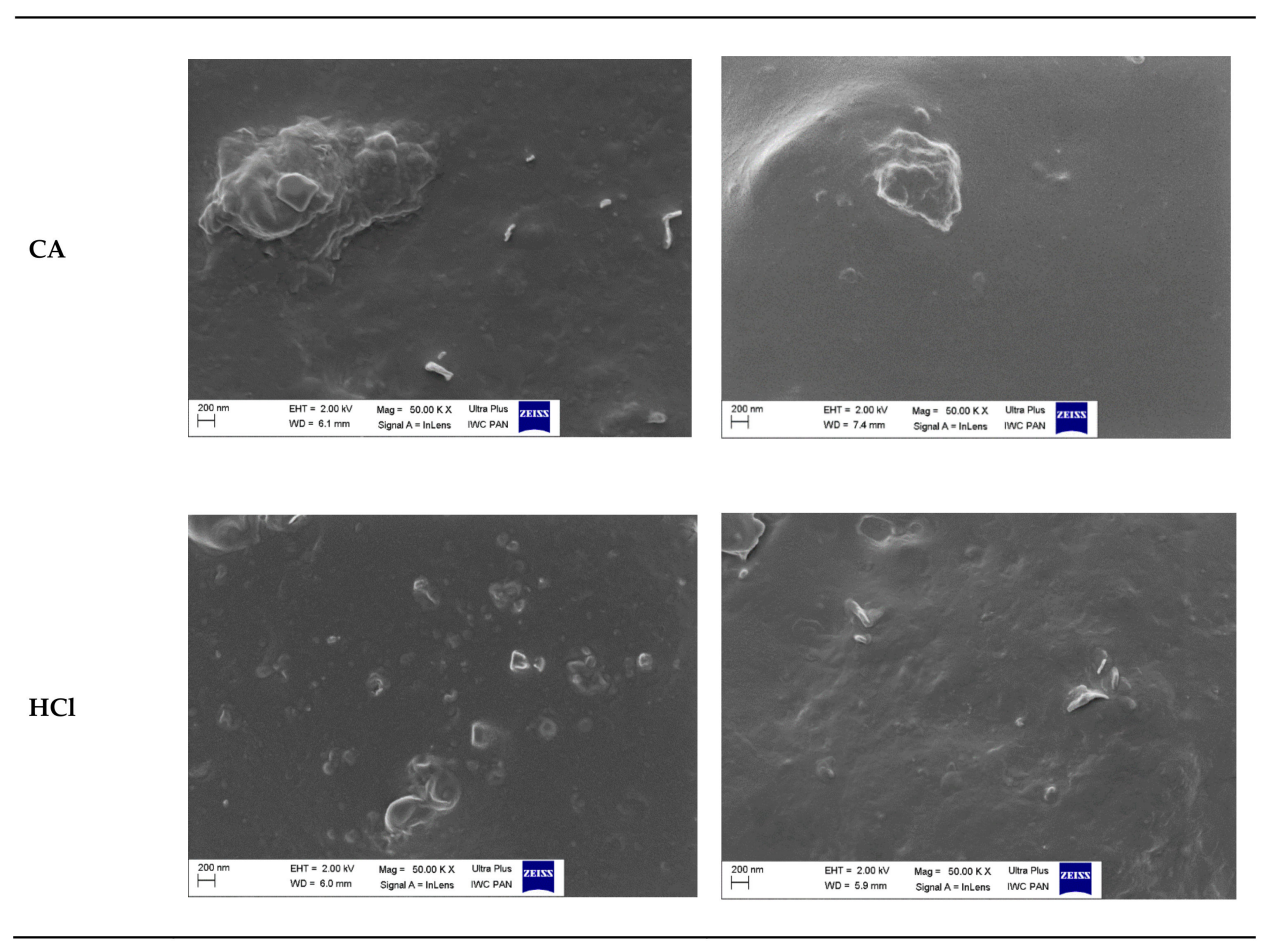
SEM images of natural rubber vulcanizates filled with phyto-ash.

Figure 10 shows SEM images of vulcanizates containing phyto-ashes obtained from cereal straw and field horsetail. The best filler dispersion was observed for composites containing additives, which were treated with hydrochloric acid during preparation (WS_HCl and HT_HCl). The filler particles were evenly distributed throughout the polymer matrix. The size of the visible agglomerates was about a dozen nanometers. Moreover, these fillers had the most porous structure and were rich in biogenic nanosilica. Slightly worse dispersion of composite components was noted for the remaining vulcanizates. In these cases, the filler clusters were much larger, their sizes reaching several micrometers. Particularly for composites filled with non-hydrolyzed additives, large aggregates and agglomerates of filler with irregular structure and size were visible. This could be due to the presence of a large amount of impurities in the filler and other compounds besides silica.

### 3.11. Mechanical Properties of Vulcanizates

**Figure 11:**
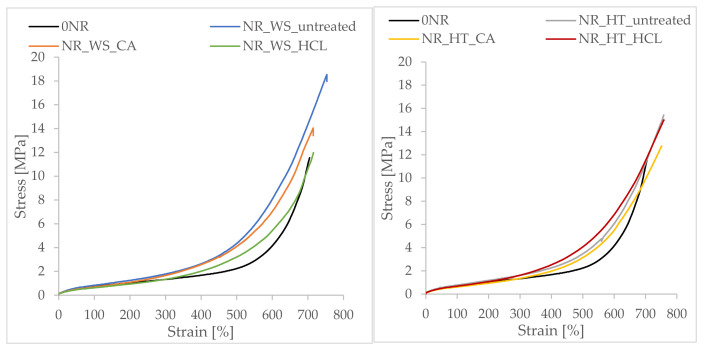
Stress–strain curves of vulcanizates filled with biofiller.

Apart from silica, phyto-ashes contain many other substances that may affect the final properties of vulcanizates. Most of such substances are found in non-hydrolyzed samples. Apart from the composition, the morphology of the silica contained in phyto-ashes also plays an important role. In order to investigate the influence of these factors, strength tests were carried out on the obtained vulcanizates. The stress–strain curves of vulcanizates recorded during the tests of static mechanical properties are illustrated in Figure 11.

The analysis of the data presented in Table 6 showed that the addition of phyto-ashes had a diversified effect on the mechanical properties of the vulcanizates. Taking into account the stresses at low strains (5%, 50%, 100% and 200%), no reinforcement of natural rubber by silica-rich phyto-ashes was observed. The values of SE100 and SE200 obtained for these composites were comparable or lower than for the reference sample. Strain induced crystallization at high elongation (strain >300%) plays a major role in the mechanical properties of NR [61]. Hence, the unfilled system obtained such high mechanical strength (approximately 11.5 MPa). The onset of crystallization is influenced by the filler content [62], as the effective filler volume fraction includes immobilized rubber, which is restrained on the particle surface because of attractive interactions between the filler and polymer chains. This layer exhibits slow dynamics. In the case of filled systems, apart from the crystallization of the elastomer, the strengthening effect of nano-additives has a significant influence on the mechanical properties. As the deformation increased (over 300%), the addition of fillers increased the stress for a given deformation in relation to the unfilled system (Figure 11 and Table 6). It was the effect of strengthening the material by creating a filler structure in the polymer matrix and a consequence of increasing the cross-linking density, especially in the case of phyto-ashes obtained from nonhydrolyzed plants. All filled vulcanizates showed an increase in tensile strength compared to the reference sample. The highest TS values were found for the NR_WS_untreated and NR_HT_untreated samples and were respectively 18.0 MPa and 15.3 MPa.

In the case of phyto-ashes obtained from hydrolyzed plants, among wheat straw, sample NR_WS_CA showed better strength, while among field horsetail, sample NR_HT_HCl. These results correspond to the previously presented and discussed analysis of the Payne effect. Moreover, these samples for individual groups showed the most extensive structure, which contributed to a slight strengthening of the material. All vulcanizates were characterized by high deformation at break. Eb values ranged from 705% for the reference sample to 715–750% for the filled systems.

### 3.12. Dynamic Mechanical Properties of NR Vulcanizates

Considering the recorded tanδ curves as a function of temperature obtained for natural rubber vulcanizates, the appearance of a peak representing the glass transition of the elastomer was observed, with the maximum determining the glass transition temperature (Tg). The type of phyto-ash used did not significantly affect the obtained Tg values, despite the fact that some of the additives used increased the cross-linking density of the vulcanizates. Elastomers are inherently materials that have a low cross-link density, unlike resins. Hence, the slight increase in cross-link density obtained for the filled composites did not significantly limit the mobility of the rubber macromolecule chains, thus the Tg value remained constant at 67–68 °C (Table 7).

**Figure 12:**
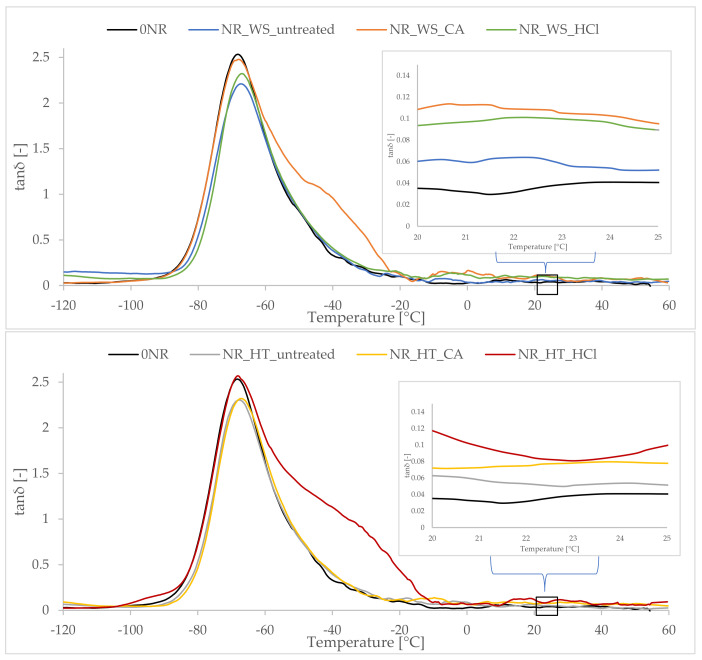
Mechanical loss factor as a function of temperature for the composites containing phyto-ash.

The loss factor (tanδ) is a measure of a material’s ability to dampen vibrations [63]. As for the lowest Tg loss coefficient, the samples containing ash from non-hydrolyzed plants, which were much stiffer than other samples, because they had a higher cross-link density, showed them. On the other hand, the vulcanizates filled with phyto-ashes were characterized by a higher tanδ in the room temperature range (including the elastic region of the rubber), which indicated better damping properties compared to the reference sample (Figure 12). In general, the tested elastomeric materials showed stable dynamic properties at temperatures of use. The values of the mechanical loss coefficients did not change significantly with increasing temperature in the rubber-elastic region.

## 4. Conclusions

The acid hydrolysis as the first step in the purification of cereal straw and horsetail with hydrochloric acid was more effective (37%, 42%) than that with citric acid (20%, 36%). In turn, the high-temperature treatment resulted in a significant reduction of the lignocellulosic material, along with the thermal degradation of organic compounds (about 90%). This process took place in three stages, and the changes in the loss of mass and its intensity resulted from different chemical composition of plants and acidification of the samples. Elemental analysis of plant materials using the ICP-OES and SEM-EDS methods showed that the non-hydrolyzed ash of both straw and horsetail contains elements such as Ca, K, Cl, S, P, Mg, Fe and, above all, large layers of silicon, which confirmed the literature assumptions. Moreover, the analysis of the infrared spectra with the Fourier transformation also confirmed the high content of silica in the obtained phyto-ashes in the tested plant materials. It is worth noting that the applied method of chemical treatment also influenced the chemical composition of the obtained bioadditives, as well as their structure, which was proved by scanning electron microscopy images.

Different morphology of the obtained phyto-ashes as well as their composition influenced the results of the vulcanization process and the course of the vulcanization curves. The viscosity of the mixtures with nonhydrolyzed samples and their increase in torque (ΔM) were significantly higher than those of the hydrolyzed samples. This was probably due to the removal of some basic compounds promoting the formation of crosslinks during the hydrolysis. The results of the crosslinking density of vulcanizates were the reflection of the rheometric properties. The addition of phyto-ashes had various effects on the mechanical properties of the vulcanizates. In the case of bioadditives obtained from hydrolyzed plants, among wheat straw, the NR_WS_CA sample showed better strength, while among field horsetail, the NR_HT_HCl sample. Moreover, these composites showed the most extensive structure, as confirmed by the analysis of the Payne effect. The tested elastomeric materials showed stable dynamic properties at temperatures of use, while the composites filled with hydrolyzed phyto-ashes showed a greater ability to dampen vibrations.

## Figures and Tables

**Table 1:** Composition of rubber mixtures.

Sample	Filler Content	NR	S	MBT	SA	ZnO
	(phr)	(phr)	(phr)	(phr)	(phr)	(phr)
0NR	0	100	2	2	1	5
NR_WS_untreated	10	100	2	2	1	5
NR_WS_CA						
NR_WS_HCl						
NR_HT_untreated						
NR_HT_CA						
NR_HT_HCl						

**Table 2:** Hydrolysis and high-temperature treatment efficiencies.

Sample	E_1_ (%)	E_2_ (%)
WS_untreated	-	95.78 ± 0.38
WS_HCL	42.89 ± 1.09	96.03 ± 0.53
WS_CA	20.32 ± 3.02	97.00 ± 0.32
HT_untreated	-	84.45 ± 0.37
HT_HCL	37.30 ± 6.43	90.43 ± 1.46
HT_CA	36.17 ± 1.00	91.84 ± 0.64

E_1_—hydrolysis efficiency; E_2_—high-temperature treatment efficiency.

**Table 3:** Content of selected elements in fillers.

Sample	Mg	Ca	Fe	Al	Si	Solid Residue (mg)
WS_untreated	1.29	4.27	0.60	0.21	0.64	15.8
WS_CA	0.08	1.73	1.09	0.30	1.31	46.6
WS_HCl	0.03	0.51	0.97	0.33	1.50	47.9
HT_untreated	2.58	13.54	0.03	0.22	1.78	10.5
HT_CA	0.22	8.23	0.06	0.25	1.13	53.2
HT_HCl	0.16	1.91	0.02	0.27	1.17	50.7

**Table 4:** Rheometric characteristic of rubber mixtures.

Sample	M_min_ (dNm)	M_max_ (dNm)	ΔM (dNm)	t_05_ (min)	t_90_ (min)
0NR	0.85	5.44	4.59	0.60	2.31
NR_WS_untreated	0.41	5.69	5.28	0.38	1.82
NR_WS_CA	0.2	4.89	4.69	0.50	2.10
NR_WS_HCl	0.15	4.46	4.31	0.65	2.63
NR_HT_untreated	0.56	5.73	5.17	0.43	1.81
NR_HT_CA	0.25	5.21	4.96	0.40	1.81
NR_HT_HCl	0.28	5.11	4.83	0.53	2.40

**Table 5:** Vulcanizates cross-linking density.

Sample	ν_e_ ×10^−5^ [mol/cm^3^]
0 NR	1.78
NR_HT_untreated	2.23
NR_HT_HCl	1.89
NR_HT_CA	1.85
NR_WS_untreated	2.37
NR_WS_HCl	1.49
NR_WS_CA	1.75

**Table 6:** Mechanical properties of vulcanizates filled with biofillers.

Sample	SE_5_ (MPa)	SE_50_ (MPa)	SE_100_ (MPa)	SE_200_ (MPa)	SE_300_ (MPa)	TS (MPa)	Eb (%)
0NR	0.18 ± 0.01	0.53 ± 0.01	0.74 ± 0.03	1.09 ± 0.08	1.48 ± 0.21	11.5 ± 0.7	705 ± 6
NR_WS_untreated	0.19 ± 0.01	0.61 ± 0.02	0.82 ± 0.01	1.22 ± 0.02	1.73 ± 0.04	18.0 ± 0.7	751 ± 1
NR_WS_CA	0.19 ± 0.01	0.50 ± 0.02	0.67 ± 0.02	1.06 ± 0.04	1.60 ± 0.09	13.9 ± 0.1	714 ± 1
NR_WS_HCl	0.17 ± 0.01	0.47 ± 0.01	0.62 ± 0.02	0.93 ± 0.03	1.33 ± 0.07	12.0 ± 0.5	715 ± 16
NR_HT_untreated	0.20 ± 0.01	0.57 ± 0.01	0.74 ± 0.01	1.17 ± 0.01	1.62 ± 0.01	15.3 ± 0.1	745 ± 18
NR_HT_CA	0.16 ± 0.01	0.44 ± 0.01	0.64 ± 0.02	0.99 ± 0.04	1.41 ± 0.05	13.4 ± 0.5	744 ± 6
NR_HT_HCl	0.17 ± 0.01	0.49 ± 0.02	0.69 ± 0.02	1.09 ± 0.03	1.64 ± 0.07	14.9 ± 0.2	737 ± 31

**Table 7:** Glass transition temperature of natural rubber vulcanizates filled with phyto-ash.

Sample	Tg (°C)
0NR	−68.0
NR_WS_untreated	−67.4
NR_WS_CA	−67.9
NR_WS_HCl	−67.1
NR_HT_untreated	−67.7
NR_HT_CA	−66.8
NR_HT_HCl	−68.2

## Data Availability

Data sharing not applicable.
